# Role of milk carbohydrates in intestinal health of nursery pigs: a review

**DOI:** 10.1186/s40104-021-00650-7

**Published:** 2022-01-05

**Authors:** Ki Beom Jang, Sung Woo Kim

**Affiliations:** Department of Animal Science, North Carolina State University, Raleigh, NC 27695 USA

**Keywords:** Intestinal health, Lactose, Milk carbohydrates, Milk oligosaccharides, Nursery pigs

## Abstract

Intestinal health is essential for the resistance to enteric diseases and for nutrient digestion and absorption to support growth. The intestine of nursery pigs are immature and vulnerable to external challenges, which cause negative impacts on the structure and function of the intestine. Among nutritional interventions, the benefits of milk are significant for the intestinal health of pigs. Milk coproducts have traditionally been used in starter feeds to improve the growth of nursery pigs, but their use is somewhat limited due to the high costs and potential risks of excessive lactose on the intestine. Thus, understanding a proper feeding level of milk carbohydrates is an important start of the feeding strategy. For nursery pigs, lactose is considered a highly digestible energy source compared with plant-based starch, whereas milk oligosaccharides are considered bioactive compounds modulating intestinal immunity and microbiota. Therefore, milk carbohydrates, mainly composed of lactose and oligosaccharides, have essential roles in the intestinal development and functions of nursery pigs. The proper feeding levels of lactose in starter feeds could be variable by weaning age, body weight, or genetic lines. Effects of lactose and milk oligosaccharides have been broadly studied in human health and animal production. Therefore, this review focuses on the mechanisms of lactose and milk oligosaccharides affecting intestinal maturation and functions through modulation of enterocyte proliferation, intestinal immunity, and intestinal microbiota of nursery pigs.

## Introduction

Swine industry has been facing with numerous challenges, including outbreaks of epidemic enteric diseases and removal of antimicrobial growth promoters in feeds which directly affect the efficiency and profitability of pig production. As a result, there have been increased efforts to investigate and find alternative nutraceuticals replacing the use of antibiotics to support intestinal health and growth of nursery pigs [1–3]. Intestinal challenges to nursery pigs are primarily caused by their immature intestinal system, which would hard to allow the feeds containing allergenic compounds from plant-based protein supplements as well as microbial infections [4, 5]. In general, sow milk is a natural source of nutrients for sucking piglets during lactation whereas the milk coproducts have been broadly used in milk replacers, creep feeds, and early weaner feeds. Milk coproducts have great potentials in feeds for nursery pigs because nutrients in milk are well balanced and highly digestible, supporting the intestinal health and growth of nursery pigs [6–8]. It has also been well documented that milk carbohydrates are most effectively utilized by nursery pigs providing energy and functional properties to support their growth and health [7, 9, 10].

Milk coproducts contain lactose as the major carbohydrate that is highly digestible compared with other carbohydrate sources in cereal grains for nursery pigs [11–13]. The main reason for high lactose digestibility would be related to early adaptation of their digestive system to maintain high lactase activity in the intestinal epithelial cells [14, 15]. In general, milk coproducts have been broadly used to supply lactose in nursery feeds. However, feeding lactose over a tolerance level would cause severe digestive problems from massive lactose fermentation in the intestine, causing negative impacts on intestinal development [16–18]. There is still a lack of updated information on the recommendation levels of lactose for nursery pigs with the changes in lean gain potential, health status, and weaning ages. Previous studies investigating an optimal lactose level in nursery feeds have shown significant variations due to changes in the genetics of pigs and management environments. However, one of the consistent results is that optimal lactose levels in feeds decrease as a pig grows [7, 10, 19].

Recent evidence suggests that animal milk and milk coproducts contain various forms of milk oligosaccharides with beneficial prebiotic effects on intestinal health and brain development in infants [20–23]. Milk oligosaccharides are non-digestible carbohydrates and the third-abundant-nutrients next to lactose and fat in animal milk [24]. These milk oligosaccharides are largely diverse and complex with their structure compared with conventional prebiotics such as galacto-oligosaccharides, xylo-oligosaccharides, or fructo-oligosaccharides which are current used in infant formula [25–28]. Once ingested, milk oligosaccharides would not be absorbed but pass through the gastrointestinal tract (GIT) and then be utilized for fermentation by intestinal microbes resulting in an increased proportion of potentially beneficial bacteria, including *Bifidobacterium* and *Lactobacillus* sp. [29–31]. Interestingly, a minimal amount of milk oligosaccharides could be absorbed into systemic circulation in young animals, possibly affecting the activation of the immune system [32–34]. Due to the diversity and specific structures of milk oligosaccharides, their mode of action for beneficial effects on young animals may be distinctively different from that of conventional prebiotics.

Therefore, there is growing interest in finding effective feeding values of milk carbohydrates for their nutritional and functional roles in the intestinal development and health of nursery pigs. This review focus on the functional role and feeding application of milk carbohydrates in modulating the intestinal health of nursery pigs.

## Lactose

Lactose is the most abundant carbohydrate in milk, which consists of galactose and glucose by β-glycosidic linkage. It can be hydrolyzed by lactase (β-D-galactosidase) secreted from epithelial cells lining villi on the small intestine. After the hydrolysis of lactose, galactose and glucose are transported to central circulatory system via Na^+^-D-glucose cotransporter 1 (SGLT1) and glucose transporter 2 (GLUT2) by facilitated diffusion across brush border and basolateral membranes of enterocytes in the intestine [35]. Once absorbed, these monosaccharides are utilized as carbon donors to generate adenosine triphosphate (ATP) in energy metabolism and assist amino acids (AA) synthesis. In particular, galactose would be converted to glucose-1-phosphate through the Leloir pathway, and then it could be used in the cellular respiration process to produce energy [36]. Despite playing such a direct role as an energy source, lactose could also be involved in AA synthesis and other potential carbon chains indirectly contributing to growth [37–39].

Although pigs have a digestive system with a relatively high tolerance level of lactose compared with other species [40], milk coproducts are minimally used in swine feeds due to the high costs compared with the use of other carbohydrates from cereal grains. Thus, feeding a proper amount of lactose in feeds is important for successful pig production, balancing the feed costs and growth of nursery pigs. Nursing piglets can get lactose from sow milk. Lactose composition in sow milk is about 3% (or 13% dry matter (DM) basis) in the colostrum and about 5% (27% DM basis) in the mature milk [9, 11, 41]. The digestive system of nursing piglets, therefore, would maintain high lactase activity [14, 15, 42]. Thus, the use of milk coproducts has been a standard practice to supply effective carbohydrate sources in nursery feeds to improve the growth performance of nursery pigs. However, the suggested optimal inclusion levels of lactose for maximal growth efficiency vary depending on studies. Therefore, a depth review is needed to understand the variability and to provide comprehensive data analysis.

### Meta-analysis to estimate lactose requirements for nursery pigs

In this review, a meta-analysis was conducted to determine the optimal dietary need of lactose for nursery pigs based on their growth performance. Data from published articles were obtained from public databases including PubMed, Science Direct, and Web of Science with criteria for filtering. For the criteria, all data from articles consisted of peer-reviewed publications. Secondly, data were obtained from animal experiments testing the effects of different levels of crystalline lactose or milk coproducts as a source of lactose. Lastly, all of the data for growth parameters, including BW, ADG, ADFI, and G:F were obtained or could be calculated from the data.

**Fig. 1:**
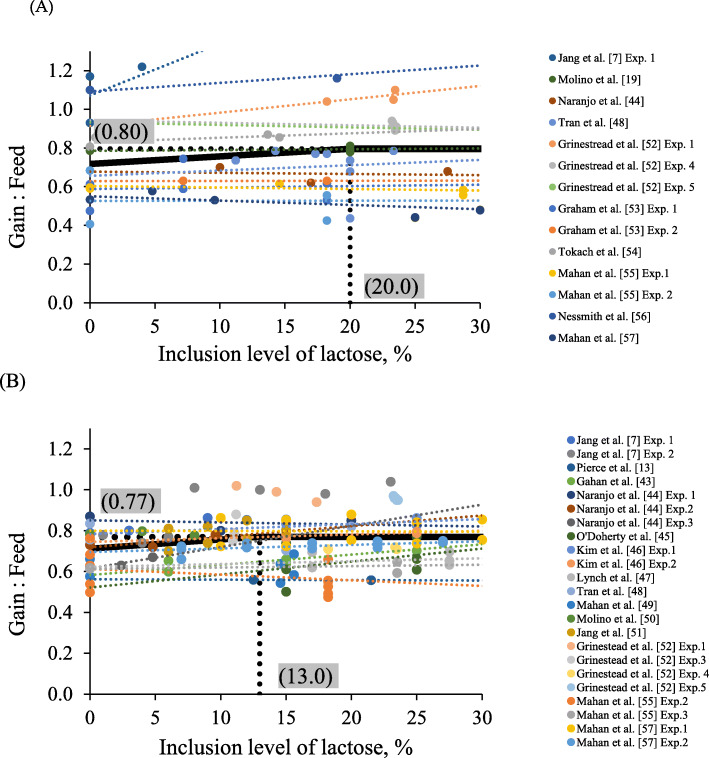
Changes in G:F of nursery pigs fed diets by inclusion level of lactose in diets during 5 to 7 kg (**A**) and 7 to 11 kg (**B**) BW using a broken-line analysis. The meta-analysis is conducted by Proc NLMIXED in SAS 9.4 to determine break-points on the regression of feed efficiency of nursery pigs calculated based on the data from 10 published studies (Phase 1 for 5 to 7 kg BW: 14 experiments) and 14 published studies (Phase 2 for 7 to 11 kg BW: 23 experiments). The breakpoints (one-slope broken-line model) were inclusion levels of lactose at 20% during 5 to 7 kg BW and 13% during 7 to 11 kg BW when G:F were 0.80 and 0.77, respectively. One-slope broken-line models; The equation for G:F during 5 to 7 kg BW was G:F = 0.80–0.39 × 10^− 2^ × *z1*, R^2^ = 0.90. The equation for G:F during 7 to 11 kg BW was G:F = 0.77–0.24 × 10^− 2^ × *z1*, R^2^ = 0.76 was; if lactose supplementation is ≥ breakpoint, then *z1* = 0; if lactose supplementation is < breakpoint, then *z1* = lactose supplementation - breakpoint

**Fig. 2:**
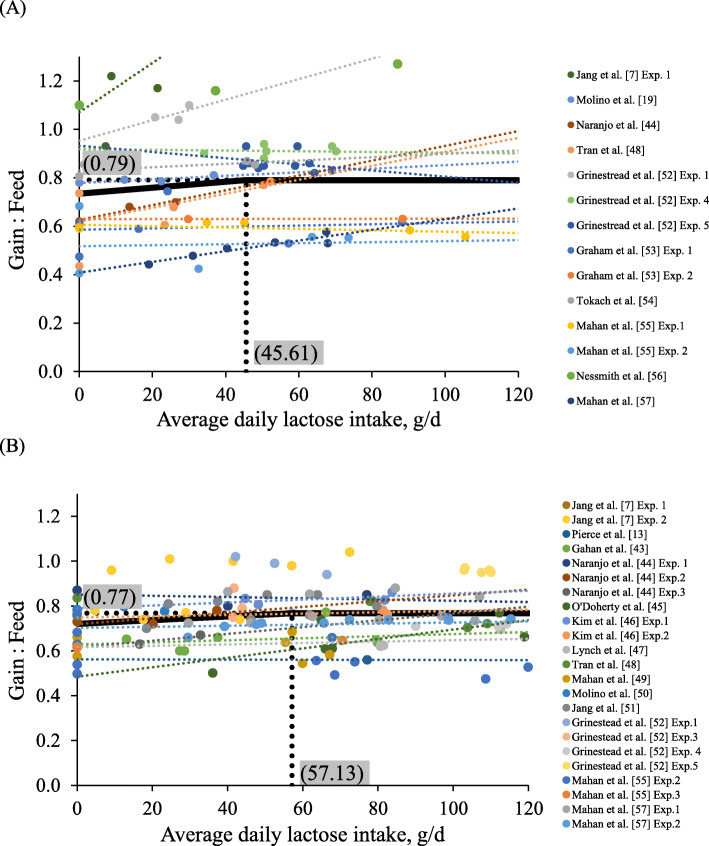
Changes in G:F of nursery pigs fed diets by average daily lactose intake during 5 to 7 kg (**A**) and 7 to 11 kg (**B**) BW using a broken-line analysis. The meta-analysis is conducted by Proc NLMIXED in SAS 9.4 to determine break-points on the regression of feed efficiency of nursery pigs calculated based on the data from 10 published studies (Phase 1 for 5 to 7 kg BW: 14 experiments) and 14 published studies (Phase 2 for 7 to 11 kg BW: 23 experiments). The breakpoints (one-slope broken-line model) were average daily lactose intake at 45.61 g/d during 5 to 7 kg BW and 57.13 g/d during 7 to 11 kg BW when G:F were 0.79 and 0.77, respectively (*P* < 0.05). One-slope broken-line models; The equation for G:F during 5 to 7 kg BW was G:F = 0.79–0.12 × 10^− 3^ × *z1*, R^2^ = 0.89. The equation for G:F during 7 to 11 kg BW was G:F = 0.77–0.83 × 10^− 4^ × *z1*, R^2^ = 0.76 was; if average daily lactose intake is ≥ breakpoint, then *z1* = 0; if average daily lactose intake is < breakpoint, then *z1* = average daily lactose intake - breakpoint

The comprehensive data provided in this review was obtained from 17 peer-reviewed publications and utilized for the meta-analysis [7, 8, 12, 19, 43–55]. The data were divided into three groups based on BW of pigs (for 5 to 7 kg BW, published in 10 papers with the 14 experiments; for 7 to 11 kg BW, published in 14 papers with 23 experiments; for 11 to 25 kg BW, published in 12 papers with 15 experiments). Daily lactose intake was calculated using average daily feed intake (g/d) multiplied by the lactose composition in each experimental diet. For statistical analysis, the inclusion level and daily intake of lactose on growth response were evaluated with nonlinear models using the Proc NLMIXED in SAS (SAS. Inc., Cary, USA). The method of nonlinear least squares used to fit the model: *Y* = *a* + *b (R-x)* + *e*. Where ‘*Y*’ is the response of feed efficiency, ‘*a*’ is the ordinate of the breakpoint, ‘*b*’ is the slope of the line when *x* < *R*, ‘*x*’ is if the inclusion level or average daily lactose intake, ‘*R*’ is the breakpoint, ‘*e*’ is a residual error among experiments, and ‘*(R − x)*’ is defined as zero when *x* ≥ *R*. Using a broken-line analysis, optimal levels of nutrient for the parameters of interest can be obtained [7, 56, 57]. Regressions were obtained between lactose levels (%) or intake (g/d) in nursery feeds and the feed efficiency of pigs. Optimal levels (breakpoints) of lactose to maximize feed efficiency were obtained at 20.0% in feeds for 5 to 7 kg body weight (BW) and 13.0% in feeds for 7 to 11 kg BW of nursery pigs (Fig. 1) or at 46 g/d lactose intake for 5 to 7 kg BW and 57 g/d lactose intake for 7 to 11 kg BW of pigs (Fig. 2), respectively. As expected, optimal inclusion levels of lactose for maximal feed efficiency decreased, whereas optimal daily lactose intakes were increased as pig BW increased. A recent review by Zhao et al. [58] demonstrated that nursery pigs need 20% lactose during d 0 to 7 after weaning, 15% lactose during d 7 to 14, and 0% lactose during d 14 to 35. Following these estimations, a previous study by Jang et al. [7] demonstrated that the growth responses to lactose supplementation was gradually decreased as pigs increased with age.

## Milk oligosaccharides

Milk oligosaccharides are the third most abundant nutrient in mammalian milk after lactose and lipids [5, 43]. Milk oligosaccharides are composed of the five monosaccharides fucose, glucose, galactose, N-acetylglucosamine, and sialic acid [59, 60]. It has also been reported that milk oligosaccharides have over 200 distinct structures with high complexity due to branches and elongated oligosaccharide structures [32]. These milk oligosaccharides contain core units having a formation of a β1–4 glycosidic linkage between galactose and glucose, and it can be further extended via β1–3 or β1–6 linkages with additional galactose or N-acetylglucosamine [61]. The core units in milk oligosaccharides could be linear or branched and it can be further linked with fucoses and/or sialic acid residues [61]. There are three major types of oligosaccharides found in milk such as neutral, neutral N-containing, and acidic oligosaccharides and classified based on the residue located at the terminal position among fucose (neutral oligosaccharides), N-acetylglucosamine (neutral N-containing oligosaccharides), and sialic acid (acidic oligosaccharides) [60].

The diversity and composition of oligosaccharides in milk are widely variable between species or period of lactation. Human milk contains the highest abundance and structural complexity, with about 20 to 25 g/L in colostrum and 5 to 20 g/L in subsequent mature milk [32]. Bode [21] described that the concentration and complexity of milk oligosaccharides in human milk have been investigated and appear to be 10 to 100 folds higher than the milk of farm animals such as cows, goats, and pigs. The concentration of bovine milk oligosaccharides are about 0.7 to 1.2 g/L in colostrum and 0.05 to 0.10 g/L in the mature milk, with about 100 identified structures, or about 15%, overlapping with human milk oligosaccharides. Accordingly, bovine milk oligosaccharides could serve as an excellent potential source for commercially available and cost-effective milk oligosaccharides in animal production compared with milk oligosaccharides from other species [60]. Dairy production and processing are equipped with sophisticated filtration systems to separate valued milk components resulting in whey permeate with high level lactose and oligosaccharides that can provide nutritional and health benefits to animal species of interest [60, 62]. Thus, nursery pigs could potentially obtain benefits from milk oligosaccharides through supplementation of whey permeate. Jang et al. [7] found that whey permeate contained about 0.4% milk oligosaccharides and showed positive effects on the intestinal health of nursery pigs when whey permeate was supplemented into their feeds. In contrast, little is known about porcine milk oligosaccharides in regards to their concentration and structural characterization in sow milk compared with human and bovine milks. There are increasing attention to the investigation of the functional properties of porcine milk oligosaccharides and their effects on the intestinal health and development of nursing piglets. Upon farrowing, intestine of piglets would initially face with lactose and milk oligosaccharides from sow milk. Previous studies have shown that the profile of oligosaccharides in porcine milk would have a higher similarity to human milk compared with bovine milk [63, 64]. This may help to define specific benefits of milk oligosaccharides to improve intestinal health and growth of nursing piglets during lactation.

Mammalian animals do not have specific enzymes to hydrolyze the milk oligosaccharides. However, milk oligosaccharides received significant attentions for their functional benefits on intestinal development and health in animals (Table 1). Milk oligosaccharides are mostly considered as prebiotics that was defined for the first time in 1995 as “a non-digestible food ingredient that beneficially affects the host by selectively stimulating the growth and/or activity of one or a limited number of bacteria in the colon, and thus improves host health” [65]. According to the International Scientific Association of Probiotics and Prebiotics (ISAPP) [66], milk oligosaccharides are also dietary prebiotics, because milk oligosaccharides are also selectively utilized by host microorganisms providing health benefits. Furthermore, milk oligosaccharides are also meeting the other criteria for prebiotics as 1) resistance to gastric pH acidity, absorption, and hydrolysis of mammalian enzymes, 2) fermentable by intestinal microflora, 3) selectively interaction with intestinal microbiota such as *Lactobacillus* and *Bifidobacterium* in the small intestine and colon [66–68]. Milk oligosaccharides can also directly or indirectly interact with enterocytes, leading to host protection by improving intestinal health [21]. Milk oligosaccharides can also protect the intestine from damages by increasing enterocyte proliferation [69, 70]. Interestingly, once arrived into the intestine, milk oligosaccharides would possibly bind to immune receptors on the innate immune cells or enterocytes, leading to the immune-modulatory and protective functions in the animal body [72, 73]. In addition, the small portion of milk oligosaccharides can also be absorbed into the enterocytes of infants by active transport, and it could possibly affect the systemic immune response [71]. Therefore, milk oligosaccharides provide a spectrum of protective and immune-modulatory functions mediated either by their prebiotic role in enriching specific beneficial microbiota or by direct interaction with immune cells.

**Table 1:** Effects of milk oligosaccharides on intestinal development and health

Model	Effects	Source	Reference
In vitro	﻿↑ Differentiation in HT-29 cells by 36% and HIEC cells by 32%	Sialyllactose	[96]
	﻿↑ Apoptosis in HT-29 cells by 300% and in HIEC cells by 200%	Neutral oligosaccharides	
	﻿ ↑ Inhibition enteropathogenic *E. coli* adhesion by 40% to HEp-2 cells	HMO	[155]
	Intestinal microbial colonization ↓ *Enterococcus* by 88%*, Streptococcus* by 89%*, Veillonella* by 42%*, Eubacterium* by 81%*, Clostridium* by 80%*,* and *E. coli* by 73%*,* ↑ *Bifidobacterium infantis.* by 20% and *Bacteroides vulgatus* by 24%	HMO	[67]
	↑ HMO consumption and growth of bifidobacterial strains by maximum 200%	HMO	[156]
	↓ Binding activity of pathogen (*Neisseria meningitidis*) to carbohydrate receptors by 80%	HMO + BMO^2^	[157]
	↓ The release of mucosal proinflammatory signals of IL-8 by 60 to 70% and IL-1β attenuated *C. jejuni* invasion by 80 to 90% ﻿↓ Acute-phase mucosal immune response by 50 to 60%	Fucosyllactose	[158]
	↑ Re-epithelialization of Ca9–22 cells by 86% ↑ *Bifidobacterium* in infant batch culture by 206% ↑ ﻿*Bacteroides* in infant batch culture by 480%	Sialyllactose	[159]
	↓ IL-8 secretion by 20% in HCT8 IECs induced by Enterotoxigenic *E. coli* infection ↓ CD14 transcription and translation cells in *E. coli* infected mice by 65%	HMO	[160]
	↑ Cell apoptosis by 20% and necrosis by 56% in Caco-2Bbe cells ↑ Cell differentiation in HT-29 cells by 25%	HMO	[161]
	↓ Adhesion of *E. coli* by 25%, *V. cholerae* by 9%, and *S. fyris* by 9% to Caco-2 cells	HMO	[148]
	↓ Adhesion of *Escherichia coli* to intestinal epithelial cells	Fucosyllactose + Sialyllactose	[162]
	↑ Binding with bacterial toxins including CTB_5_, HLTB_5_, Stx1B_5_, Stx2, TcdA2 and TcdB1 with ranging from 600 to 15,000 M^− 1^	HMO	[152]
Human	↓ Significantly frequency of diarrhea in infants	Fucosylated oligosaccharides	[163]
	↑ *Actinobacteria* and *Bifidobacterium* ↓ Firmicutes and Proteobacteria in fecal microbiota	Fucosyllactose and lacto-N-neotetraose	[164]
	↑ Firmicutes in the feces of infants by 250%, ↓ Enterobacteriales infants by 50%,	HMO	[165]
Rodent	↑ Abundance of *Lactobacillus* by 30% in cecal and colonic microbiota ↓ mRNA levels of colonic tumor necrosis factor-α (*TNF-α*) by 70% in cecum and colon	BMO	[166]
	↓ Gene expression of *TNF-a* by 85%, *IL-6* by 50%, and *IL-1ß* by 70% in colon of mice ↑ Gene expression of *TGF-ß* by 90% and occludin by 95% in colon of mice	Fucosyllactose	[167]
Porcine	↑Glutamate dehydrogenase by 44% in the serum	Sialyllactose	[168]
	↓ Diarrhea occurrence by 32% induced by rotavirus ↑ Dry matter contents by 5% of colonic contents ↑ IFN-γ by 25% and Il-10 by 30% in the ileum ↑ Relative abundance of *Lachnospiraeae* as butyrate-producing bacteria by 100% in colon	HMO	[169]
	﻿↑ Length of villi by 16% in the ileum ↓ BW loss by 80% induced by E.coli challenge	﻿Fucosyllactose	[170]
	﻿↑ Il-12 by 300% in the ileum, short chain fatty acids production by 43%, and expression of TLR4 by 40% in the colon	HMO	[171]

^1^Human milk oligosaccharides

^2^Bovine milk oligosaccharides

## Mode of action of milk carbohydrates benefiting immature intestine of nursery pigs

Previous studies indicated that milk carbohydrates played functional roles in intestinal health of nursery pigsby regulating nutrient metabolism, immune response, enterocyte proliferation, and intestinal microbiota [7, 74]. However, the functional importance of milk oligosaccharides has not been discussed thoroughly, whereas the role of lactose has been highlighted well. Therefore, the major objective of this section is to review the published data focusing on the functional effects of both lactose and milk oligosaccharides on intestinal development and health in nursery pigs.

Recently, increasing supplemental levels of milk carbohydrates in feeds improved the growth performance of nursery pigs during the early postweaning period [7, 10]. The improvement in growth of nursery pigs fed with dietary lactose could be corresponding to the change of lactase activity by growth. Ekstrom et al. [15] reported that the lactase activity in the small intestine of nursery pigs was linearly decreased by 80% from the birth to 6 weeks of age. Previous studies demonstrated that the reduction of lactase activity could be related to an increased inflammatory response in the intestinal epithelium of the small intestine after weaning [75, 76]. Pié et al. [75] showed that the lactase activity was decreased by 60 to 80% in the entire small intestine in nursery pigs for 8 d after weaning. In particular, proinflammatory cytokines such as interferon-gamma (IFN-γ), interleukin-1beta (IL-1β), and tumor necrosis factor-alpha (TNF-α) produced for immune activation could reduce the production of digestive enzymes in the intestine [77, 78]. According to Jang et al. [7], increasing supplemental levels of whey permeate from 0 to 18.75% as sources of lactose and milk oligosaccharides linearly reduced the lactase activity by 60% compared with 0 whey permeate with increased IL-8 production in the jejunum of nursery pigs during 7 to 11 kg BW. This may be related to immunomodulatory functions of milk oligosaccharides leading to immune activation by binding to the receptors on immune cells or enterocytes in the jejunum. Another possible action could be associated with the lumen pH changes in the small intestine by milk carbohydrates [43] because it has been shown that lactose supplementation induced an increased lactic acid from intestinal fermentation, reducing the luminal pH. This could lead to a favorable condition against pathogenic bacteria [16, 79, 80].

**Fig. 3:**
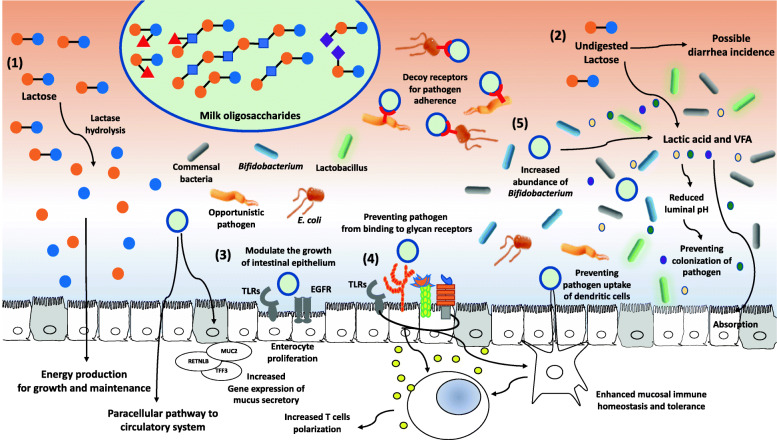
Overview of the possible functions of milk carbohydrates on the intestine of nursery pigs. Lactose and various type of milk oligosaccharides could improve the intestinal maturation and health of nursery pigs through positively modulating enterocyte proliferation, intestinal immune response, and microbiota. (1) Lactose would be hydrolyzed by lactase producing galactose and lactose, which are utilized to produce energy for the growth and maintenance of nursery pigs. (2) lactose at optimal level could induce a favorable condition against pathogenic bacteria by microbial fermentation of saccharolytic bacterial groups, including *Lactobacillus* and *Bifidobacterium,* leading to preventing pathogen colonization in the intestine of nursery pigs. In addition, (3) milk oligosaccharides could modulate the growth of intestinal epithelium through binding to toll-like receptors (TLRs) and epidermal growth factor receptors (EGFR) on the enterocytes. (4) Milk oligosaccharides can increase gene expression of mucus secretion and prevent the pathogen from binding to intestinal immune-related receptors including TLRs and various types of glycan receptors, leading to enhanced mucosal immune homeostasis and tolerance. (5) Milk oligosaccharides can prevent pathogen colonization by increased abundance of *Bifidobacterium* and production of lactic acid and volatile fatty acids (VFA)

Supplementation of milk oligosaccharides can be one of the ways to improve the utilization of lactose in the intestine of nursery pigs. In the studies using a human model, supplementation of prebiotics could mitigate the adverse effects of lactose intolerance in human adults [17, 81, 82]. When the secretion and activity of lactase in the small intestine are not sufficient and cannot hydrolyze enough lactose, an undigested portion would be fermented by colonic bacteria [83]. However, when the amount of undigested lactose is over the capacity of colonic fermentation, it would increase the occurrence of diarrhea by increased osmotic trapping of water and abnormal intestinal motility, including bloating and flatulence leading to abdominal pain and diarrhea [84, 85]. Milk oligosaccharides may help increase the capacity of intestinal fermentation of lactose by modulating the commensal intestinal bacterial population increasing beneficial microorganisms [68, 86]. Previous studies have shown that supplementation of prebiotics could reduce the symptoms of lactose intolerance by adaptive shifts in intestinal microbiota [17, 82, 87]. Therefore, supplementation of milk coproducts providing both lactose and milk oligosaccharides in nursery feeds would be beneficial for the intestinal maturation and health of nursery pigs compared with the use of crystalline lactose only. According to Jang et al. [7], supplementation of whey permeate in starter feeds improved intestinal development and growth of pigs in the early post-weaning period, indicating that supplementation of whey permeate could have positive effects on preventing jejunal dysfunction in nursery pigs at 7 to 11 kg BW. It was also reported that milk oligosaccharides could prevent pathogenic invasion, facilitate the establishment of intestinal microbiota, improve intestinal development, and stimulate immune activation [74]. These observations have significantly increased research interest in understanding interactive functional roles and nutritional importance of milk carbohydrates in intestinal maturation and health of nursery pigs (Fig. 3).

### Enterocyte proliferation in nursery pigs

Intestinal enterocytes are intensively proliferated in crypts after birth and during the postweaning period to develop the epithelial structure and recover the epithelial structure from damages by weaning stress. Thus, crypt cell proliferation has been used as a marker showing the status of intestinal maturation, development, or maintenance of pigs. According to Jang et al. [88], the proportion of proliferating cells in a jejunal crypt of neonatal piglets was near 50%, and then the proportion was decreased by 17% on d 18 of lactation. In addition, weaning stress is shown to negatively influence physiological functions and morphology of enterocytes in nursey pigs, causing increased apoptosis, proliferation, and differentiation that are critical in recovery, development, maintenance of intestinal epithelium [89–91]. According to Duarte et al. [92], the oral challenge with enterotoxigenic F18+ *Escherichia coli* to nursery pigs increased the proliferation of enterocytes by about 20% in the jejunum.

Milk oligosaccharides have been known that it can mainly have two important potential functions: 1) enhancement of growth in beneficial bacterial communities and 2) prevention of pathogen attachment to the intestinal epithelial [93]. In addition, recent papers showed that milk oligosaccharides could beneficially modulate the enterocyte proliferation in the immature intestine of the pigs. Wang et al. [94] demonstrated that milk oligosaccharides increased enterocyte proliferation by 60 to 80% in the ileum of mice after the hypoxia exposure for 3 d. Wang et al. [69] further demonstrated that milk oligosaccharides could restore enterocyte proliferation in the ileum of mice challenged with hypoxia and cold stress. Hester et al. [95] also reported that high doses of milk oligosaccharides including fucosylated-oligosaccharides, lacto-N-enotetaose, and sialylated-oligosaccharides reduced the stimulated-cell proliferation by the incubation with nucleotides in the human intestinal epithelial cells (FHs-74 Int). In a normal condition, Holscher et al. [70] showed that incubation with milk oligosaccharides for 72 h reduced the enterocyte proliferation by about 10% in HT-29 culture and by about 25% in Caco-2Bbe cell culture. Kuntz et al. [96] also showed sialylated-oligosaccharides reduced the proliferation in HT-29 and Caco-2 cell cultures. However, there is limited information in porcine studies demonstrating the effects of milk oligosaccharides on intestinal maturation and enterocyte proliferation in the small intestine. Jang et al. [7] recently showed the increasing supplemental whey permeate as sources of lactose and milk oligosaccharides linearly increased the crypt cell proliferation in the jejunum of nursery pigs.

A possible mechanism for milk oligosaccharides improving intestinal health and development would be related to their direct binding to relevant receptors on the enterocytes. Previous studies showed that milk oligosaccharides possess the ability to bind with toll-like receptors family (TLRs) and epidermal growth factor receptors (EGFR) on the cell membrane of the enterocytes, effectively modulating TLR and EGFR signal pathways which are associated with cell proliferation [94, 97, 98]. It has been known that the various TLRs and EGFR could play a role in intestinal maturation and development including enterocyte proliferation, apoptosis, migration, and immune response in the intestine [99–102]. Following a mucosal injury from viral or bacterial invasions, stress, or inflammatory response, the damage-associated molecular patterns (DAMPs), which are signal molecules released during cell injury, could be recognized by TLRs and the various signaling pathways in the intestine, resulting in an increase of epithelial cell proliferation involved in regeneration [101]. Wang et al. [69] reported that milk oligosaccharides reduced the expression of TLR4 by about 50% and the enterocyte proliferation by about 40% in the ileum of mice challenged with hypoxia and cold stress on d 3 after the challenge. Good et al. [102] also reported that breast milk significantly could reduce the TLR4 signaling with activation of the EGFR in the enterocytes of mice treated with LPS challenge. Thus, milk oligosaccharides may inhibit pathogens from binding to these receptors, and then modulate the TLR and EGFR signals in the immature intestine, leading to an increase in proliferation.

### Immune responses in the immature intestine of pigs

Oligosaccharides have been shown to exert various effects on the immune system and potentially modulate immune responses [103]. Recently, functional roles of prebiotic oligosaccharides in the intestine have been increasingly investigated [104, 105]. Among oligosaccharides, milk oligosaccharides have been shown to have functional roles influencing the development of the immune system in the immature intestine of pigs [106]. Several studies have demonstrated that the effects of milk oligosaccharides on innate immunity [107]. In vitro experiments have shown that administration of sialylated-oligosaccharides induced greater bacterial clearance in mice infected with *Pseudomonas aeruginosa* strain K and promoted receptor-mediated endocytosis and phagocytosis [108]. Furthermore, milk oligosaccharides could easily conjugate with various receptors and oligopeptide carriers in immune cells resulting in decreased virus-receptor interactions [109, 110]. Obelitz-Ryom et al. [111] also reported that the preterm piglets fed milk oligosaccharides had significantly improved the phagocytic activity of neutrophils isolated from blood. These data indicate that milk oligosaccharides could be a strong immunomodulatory agent in nursery pigs.

Newborn piglets would have a relatively low immune capacity with low numbers of leukocytes and immune-modulatory components [112, 113]. Piglets do not have sufficient defense ability due to undeveloped immune cells. In addition, weaning stress could potentially cause the intestinal dysfunction of nursery pigs during post-weaning period [114]. Thus, the neonates would take up to 6 weeks to develop a stable immunity in nursery pigs [114]. Therefore, at the first 2 to 4 weeks of life, pigs are susceptible to various types of stresses and diseases in which the intestinal immunity is not mature. The intestine has an important function as an immune barrier possessing up to 70% of immune cells in an animal body [115, 116]. As the first line of defense, intestinal barrier function is considered innate immunity. Firstly, a layer made of mucin and mucus glycoproteins could be a part of the intestinal barrier. It can act as a physical barrier between the lumen and intestinal epithelium. Milk oligosaccharides may affect the function of goblet cells in the intestine. According to Cheng et al. [117], milk oligosaccharides, especially, 2′-fucosyllactose, 3′-fucosyllactose, and lacto-N-triaose II, could enhance the mucus barrier function through increased the gene expression of mucus secretion including *MUC2*, *TFF3*, and *RETNLB* genes in the enterocytes under inflammatory and stress conditions. It is important to note that the structures of mucin glycans are similar to milk oligosaccharides. It could be further hypothesized that milk oligosaccharides modulate intestinal barrier functions by modulating the composition of intestinal microbiota [118]. Therefore, it could indicate that milk carbohydrates potentially support the intestinal barrier by promoting intestinal mucus secretion.

Secondly, milk oligosaccharides could effectively modulate the immune functions and ligand specialties through direct interaction with the various type of pattern recognition receptors such as multiple classes of lectins and TLRs in the intestinal epithelium or immune cells [33]. Previous studies have shown that milk oligosaccharides could be involved in the expression of various TLRs that could bind to the surface molecules of pathogens in the intestine. He et al. [119] showed that milk oligosaccharides reduced mRNA level of the cluster of differentiation (CD) 14 by 40% in T84 intestinal epithelial cells treated for 48 h and then challenged by LPS compared with the control group. The CD14 is engaged in TLR-4 signaling and is also a receptor enabling the recognition of the gram negative pathogenic bacteria on enterocytes under LPS-induced challenge. Milk oligosaccharides could suppress the expression of TLR4 by mediating the maturation of dendritic cells [72]. It could be supported by Zhang et al. [97], showing that milk oligosaccharides could inhibit the interaction between LPS and TLR4 by reducing pro-inflammatory cytokines including IL-6 and TNF-α in the ileum exposed to hypoxia and cold stress, leading to an increased 25% survival rate of rats.

The family of glycan receptors such as c-type lectins, galectins, selectins, and siglecs could bind to milk oligosaccharides on intestinal epithelium and immune cells [33]. It has been known that lectin receptors are glycan-binding proteins related to immune response and cell recognition [120, 121]. Xiao et al. [72] showed that milk oligosaccharides could mediate the interaction with dendritic cell-specific intercellular adhesion molecule-3-grabbing non-integrin (DC-SIGN) receptors that are c-type lectins involved in the regulation of the immune response in dendritic cells. In contrast to c-type lectins, milk oligosaccharides can also directly affect the interaction of galectins, which are receptors on T cells or enterocytes, leading to immune regulation of T cell functions [122]. He et al. [123] also reported that milk oligosaccharides increased the T-helper 1 (Th1) cells polarization with improvements in the balance of Th1 / Th2 lymphocytes in the intestinal mucosa, indicating that milk oligosaccharides may induce the maturation of immune response through enhancing the cell-mediated immune response to pathogen infection in the intestine. Nursery pigs are susceptible to intestinal inflammation caused by stress, diseases, or viral infections due to the immature development and maturation in their intestine [124, 125]. According to previous studies, milk oligosaccharides could mediate the immune receptors on immune cells and enterocytes under stressful conditions by reducing the expressions of TLRs and lectin receptors [33, 126]. Therefore, milk carbohydrates could positively support the intestinal immune response of nursery pigs by interacting with immune receptors on the intestinal epithelium.

### Microbiota in the immature intestine of nursery pigs

The composition and diversity of intestinal microbiota continue to change as a pig grows [127, 128], which can also be affected by environmental factors and dietary factors [129]. Previous studies showed that *Lactobacillus* and *Bifidobacterium* generally dominate the intestine of breast-fed animals [130–132]. Interestingly, the microbial fermentation by the saccharolytic bacterial groups, including *Lactobacillus* and *Bifidobacterium,* in the intestine would induce the production of short-chain fatty acids, which could affect the intestinal environment favorable to microorganisms reducing luminal pH [80, 133]. According to Gahan et al. [43], increasing the supplemental levels of lactose from 60 to 250 g/kg in feeds linearly decreased the fecal pH of nursery pigs by 11% on d 15 after weaning. The reduced local luminal pH could help inhibit the colonization of pathogens living in the intestinal lumen, feces, or mucosa [134, 135]. Zhao et al. [58] also reviewed that dietary lactose could be fermented by intestinal microbiota resulting in the production of lactic acid and short-chain fatty acids, conferring prebiotic-like effects on intestinal microbiota. Previous studies have also shown that supplementation of dietary lactose resulted in modulation of the abundance of specific microflora, including *Lactobacillus* [7, 136], *E. coli* [45], and *Bifidobacterium* [137].

Frese et al. [138] also showed that milk oligosaccharides could help establish the intestinal microbiota in nursery pigs. Jang et al. [7] reported that increasing the supplemental levels of whey permeate from 0 to 18.7% increased the abundance of *Bifidobacteriaceae* by 41% and showed the quadratic effects on the abundance *Lactobacillaceae* showing increasing by 31% at 13% whey permeate in the jejunum of nursery pigs during 7 to 11 kg BW. It could indicate that milk carbohydrates could positively modulate the mucosa-associated microbiota of nursery pigs. In addition, these saccharolytic bacterial groups could effectively utilize milk carbohydrates, resulting in the production of abundant short-chain fatty acids including butyrate and lactate mediated by *Bifidobacterium* and *Bacteroides* groups [139, 140]. Short-chain fatty acids have been shown to be essential energy sources for intestinal development and effective immune responses [130, 131]. Specific enzymes such as fucosidase and neuraminidase that hydrolyze milk oligosaccharides have been found in saccharolytic bacteria and these enzymes have specific functions in microbial growth and short-chain fatty acid production [140, 141]. However, so far, information on these potential beneficial impacts of milk oligosaccharides on intestinal fermentation has been elucidated through clinical trials mainly focusing on the colon in humans or in vitro studies for pigs. Pigs could effectively utilize the milk oligosaccharides for intestinal microbial fermentation due to enormous differences in the relative size of the intestine compared with human. Pigs have well-developed cecum where intestinal microbiota would ferment a part of carbohydrates before entering the colon and their cecum is significantly larger than humans [142]. It has also been known that the contribution of short-chain fatty acids to maintenance energy requirement through microbial fermentation is about 10% in humans and 20% in pigs [143, 144]. Therefore, milk oligosaccharides could display great potentials to be effectively for the production of short-chain fatty acids by microbial fermentation in the intestine of nursery pigs.

In addition, milk oligosaccharides could provide antiadhesive effects on intestinal pathogens, which use cell surface glycans to recognize and bind to the target cells, leading to pathogenesis [145, 146]. This may be due to milk oligosaccharides possessing structural similarities to cell surface glycoconjugates utilized by intestinal microbiota [145]. Additionally, it has also been known that oligosaccharides can be involved in cell recognition and signaling, specifically in microbial adhesion and microbial interactions between the intestinal epithelial cells [147]. According to previous studies, milk oligosaccharides could not only inhibit binding of the pathogens such as *E. coli* [148–150], *Helicobacter* [151], and *Salmonella* [148], and microbial enterotoxins [152], but also significantly promote the adhesion of *Bifidobacteria* to intestinal epithelial cells [153, 154]. Walsh et al. [68] demonstrated that milk oligosaccharides-containing products could potentially increase the residence time of probiotic bacteria in the intestine leading to improved or sustained intestinal health.

## Conclusion

Milk carbohydrates are essential to support the growth and intestinal health of the pigs, especially during early stage of life when the pigs are vulnerable from enteric diseases or stressful conditions negatively affecting the intestinal health. Although lactase activity is high in their early life, it is important that feeding proper amounts of lactose in early weaner feeds because if feed too high, it can cause abnormal intestinal functions from extensive lactose fermentation, unbalanced osmosis, or abnormal motility. Based on the meta-analysis of published data, it would be recommended that pigs at 5 to 7 kg BW (typical early weaner feeds) need 20% lactose (or 45.6 g/d intake), pigs at 7 to 11 kg BW (typical pre-starter feeds) need 13% lactose (or 57.1 g/d intake). Milk carbohydrates as bioactive compounds play a critical roles in improving intestinal maturation and health of nursery pigs through positively modulating enterocyte proliferation, immune response, and microbiota. Milk oligosaccharides promote the utilization of lactose by positive modulation of immune responses and composition of intestinal microbiota in young pigs. Based on the current knowledge and research, it is warranted to further investigate whether milk carbohydrates can have specificity of prebiotic effects depending on the types, ratio, or combination status.

## Data Availability

All data generated or analyzed during this study are available from the corresponding author upon reasonable request.

## Abbreviations

AA: Amino acids
ATP: Adenosine triphosphate
BW: Body weight
CD: Cluster of differentiation
DAMPs: Damage-associated molecular patterns
DC-SIGN: Dendritic cell-specific intercellular adhesion molecule-3-grabbing non-integrin
DM: Dry matter
EGFR: Epidermal growth factor receptors
GIT: Gastrointestinal tract
GLUT2: Glucose transporter 2
HMO: Human milk oligosaccharides
BMO: Bovine milk oligosaccharides
IFN-γ: Interferon-gamma
IL: Interleukin
ISAPP: International scientific association of probiotics and prebiotics
LPS: Lipopolysaccharides
SGLT1: Na^+^- D-glucose cotransporter 1
Th: T-helper cells
TLR: Toll-like receptors
TNF-α: Tumor necrosis factor-alpha
